# Supplementary material for: The Structure and Organizations of ICHD-3 Differential Diagnoses through DiffNet: A Pilot Study
Source: Diagnostics (Basel). 2022 Oct 25;12(11):2589. doi: 10.3390/diagnostics12112589 (PMC9689765; doi:10.3390/diagnostics12112589)
Supplement: Supplementary file 1 [file diagnostics-12-02589-s001.zip › diagnostics-1941791-supplementary/Examples.pdf]

Examples of Differential Diagnosis Based on DiffNet:

Migraine without aura:

1. calcitonin gene-related peptide (cgrp)-induced headache
2. pure menstrual migraine with aura
3. non-menstrual migraine without aura
4. headache attributed to moyamoya angiopathy (mma)
5. episodic syndromes that may be associated with migraine
6. angiography headache
7. headache attributed to an intracranial endarterial procedure
8. triptan-overuse headache
9. menstrually related migraine with aura
10. status migrainosus
11. nitric oxide (no) donor-induced headache
12. vestibular migraine
13. typical aura without headache
14. probable migraine without aura
15. headache attributed to fasting
16. chronic tension-type headache
17. pure menstrual migraine without aura
18. menstrually related migraine without aura
19. cardiac cephalalgia
20. infantile colic
21. chronic migraine
22. migraine
23. medication-overuse headache (moh)
24. histamine-induced headache
25. abdominal migraine
26. frequent episodic tension-type headache
27. migraine aura-triggered seizure
28. migrainous infarction
29. non-menstrual migraine with aura
30. phosphodiesterase (pde) inhibitor-induced headache
31. headache attributed to cerebral venous thrombosis (cvt)
32. probable migraine

Infrequent episodic tension-type headache

1. probable infrequent episodic tension-type headache
2. nitric oxide (no) donor-induced headache
3. infrequent episodic tension-type headache associated with pericranial tenderness
4. infrequent episodic tension-type headache
5. probable migraine
6. infrequent episodic tension-type headache not associated with pericranial tenderness

Frequent episodic tension-type headache

1. probable frequent episodic tension-type headache
2. headache attributed to psychiatric disorder
3. frequent episodic tension-type headache associated with pericranial tenderness
4. nitric oxide (no) donor-induced headache
5. chronic tension-type headache
6. medication-overuse headache (moh)
7. migraine without aura
8. frequent episodic tension-type headache
9. tension-type headache (tth)
10. probable migraine
11. frequent episodic tension-type headache not associated with pericranial tenderness
