# Supplementary material for: The Structure and Organizations of ICHD-3 Differential Diagnoses through DiffNet: A Pilot Study
Source: Diagnostics (Basel). 2022 Oct 25;12(11):2589. doi: 10.3390/diagnostics12112589 (PMC9689765; doi:10.3390/diagnostics12112589)
Supplement: Supplementary file 1 [file diagnostics-12-02589-s001.zip › diagnostics-1941791-supplementary/supplementalMaterial_The Technical Addendum.pdf]

**Supplemental material - (The Technical Addendum)**

The study of sets and their subsets relations belong to the mathematical discipline of order theory and lattice theory.[19] The theoretical underpinning of this project is the application of these theory to the realm of differential diagnosis. In this technical addendum we will identify the mathematical and theoretical basis of our project.

First, let us introduce two closely related mathematical concepts, partial ordered sets (called posets for short) and preorder.[20,21]

**Definition: Partial Preorder:**

Given P and elements a, b in P, then P is a partial preorder if:

1. $a \leq a$ for all a in P
2. If $a \leq b$ and $b \leq c$ then $a \leq c$.

**Definition: Strict Preorder:**

1. ($a < a$) is false for all a in P
2. If $a < b$, and $b < c$, then $a < c$.

With the above two definitions, we can introduce partial ordered set, often called "poset" for short.

**Definition:**

Partial ordered sets is a set $(P, \leq)$ such that P is a set and $\leq$ is a partial preorder on P.

Of note, any poset can be graphically represented using Hasse Diagram where set/subset relations are described by lines representing set/subset relationships in a downward fashion. We have avoided this presentation for clarity for the non-technical reader.

**A Potential Topological Implication:**

It is well established that posets is isomorphic to a topological space called the Alexandrof Space. Therefore, an implication of our project is that the subset relations between differential diagnoses may, with slight modification, be interpreted topologically.[22] To our knowledge, this is only the second incidence of interpreting clinical entities topologically. (The first one was proposed by the author.)[23] This correlation to topology can be exploited in future endeavors of translating theorems proved in Alexandrof Topology to the subset relation of differential diagnoses.

**Joint Semilattice:**

As a poset, our project turns differential diagnosis sets into a join-semilattice if we allow all 51 super-set as well as singletons to be subsumed under a common heading. (We can call this 1, as per lattice theory parlance.) Consider the following definition:

**Definition:**[24]

Let $(P, \leq)$ be a poset and x and y elements of P. Then meet of x and y, denoted $x \wedge y$, is the following, if it exists:

$$\max\{\text{for all } w \text{ in } P: w \leq x, w \leq y\}$$

Similarly, the join of x and y, denoted $x \vee y$, is the following, if it exists:

$$\min\{\text{for all } z \text{ in } P: x \leq z, y \leq z\}$$

In other words, meet is the maximum lower bound and join is the minimum upper bound between x and y. We can then define semilattice as the following: If every pair in x, y in P has a

join then it forms a join semilattice. Similarly, if every pair x, y in P has a meet then it forms a meet semilattice.[25,26]

We can prove computationally that our 51 poset is indeed such a construction. (We include this as Supplementary Material 1.) Since the join of any two singleton is 1 and the join of any singleton with any elements of the 51 poset semilattice is also 1, we can then confirm that our construction forms a join semilattice.

**"Addition" of Differential Diagnosis as Algebraic implication:**

Any join semi-lattice can be viewed as a idemopotent and commutative monoid under "join" operation, therefore our construction is a monoid.[27] We can view the "join" operation as therefore a sort of "addition" for differential diagnoses. For example, given differential diagnosis a and b, we can always find a differential diagnosis set that includes both as its subset. For example, the "addition" (under "join" operation) of differential sets "visual snow" (31v) and "retinal migraine"(31r) would be the differential diagnosis set of "migraine"(31).

More interestingly is investigating whether "meet" exists in our poset; if addition of differential diagnosis through "join" is considered "expansion" of differential, then addition of differential through "meet" would be consider the narrowing of differential. We screened all possible non-trivial lower bound for meet as part of our project. (Trivial here means that if a = meet(a, b) then it is trivial.) Result is presented in Table 4. Unfortunately, the set of meet is not surprising, as the majority of meet are actually obvious connections between two diseases.

**Categorification and implication for diagnostic rule out:**

Our construction of subset relations between differential diagnosis can be easily turned into a strict poset relationship if we uses $\leq$ rather than $<$. If we allow for such a simple modification of

our project, then our construction for differential diagnosis conveniently forms a mathematical category. This is due to the fact that any poset forms a thin category. We introduce both of these definitions below:

**Definition**:[28,29]

A category A consists of:

1. A collection of objects, denoted obj(A)
2. For each a, b in obj(A), a collection of maps, also called arrows or morphism, from a to b, denoted $hom_A(a, b)$. Often written as hom(a, b). In other words, per MacLane:

   $hom_A(a, b) = \{f \mid f \text{ is an arrow } f: a \text{ -> } b \text{ in } A\}$

3. For each a, b, c in hom(A), a function hom(b, c) × hom(a, b) -> hom(a, c) called composition.
4. For each element a of obj(A), an element $1_A$ in hom(a, a), called identity on a.

The above data must satisfy the following:

5. Associativity: for each f in hom(a, b) and g in hom(b, c) and h in hom(c, d) then (h . g) . f = h . (g . f)
6. Identity: for each f in hom(A, b), f . $1_A$ = $1_B$ . f.

A thin category is a category with at most one morphism. It is also called a posetal category.

**Definition**:[28,29]

Let C, B be two categories. A functor is defined by two functions: and object function T and an arrow function, also written as T, satisfying the following:

1. Object function T assigns an each object c in C to an object Tc in B.

2. Arrow function assigns each arrows f in hom(c, c’) in C to an arrow Tf in hom(Tc, Tc’) of B.
3. $T(1_c) = 1_{TC}$
4. T(g . f) = Tg . Tf, whenever composition is defined in C for g and f.

Establishing functorial relationships between different categories allows us to describe diagnostic rule outs. For example, consider the case where “headache secondary to TIA” is ruled out by a diagnostic intervention. We can construct a new poset from our existing one by simply taking out “headache secondary to TIA” from all of its elements. (Represented by the notation “\”.) One can show that the new poset constructed through this rule out retains the exact same set/subset relationship.

Diagnostic rule out of “headache secondary to TIA” from our original poset is therefore a functor in the following way 1) the functor maps differential set in the original poset to a differential set without the “headache secondary to TIA” and 2) the functor maps the function ≤ to ≤ in the new poset. That is, $F(a \leq b) = Fa \leq Fb$. Notice that $F(f . g) = F (x < z) = (x \setminus b) \leq (z \setminus b)$ for any f: x-> y and g: y -> z, where b = “headache secondary to TIA”. Also $F(f) . F(g) = (x \setminus b \leq y \setminus b) \leq ( y \setminus b \leq z \setminus b) = (x \setminus b) \leq (z \setminus b)$. Therefore the functor relationship is satisfied.

The importance of being able to construct such a functor lies in the ability for us to write addition as well as diagnostic rule out as a formula. Consider an initial differential diagnosis, a, we can expand this diagnosis by adding another diagnosis, b, via join, and then rule out a condition C. This sequent can be written as $C(a \vee b)$.

**Theoretical Conclusion and Future directions:**

Our project provides a potential theoretical foundation for the exploration of orders and hierarchy in clinical science apart from ordering based on pathophsyiology. In other words, while ICHD3 classification classifies headache disorders pathophsyiologically, our schema establishes a foundation for classification of differential diagnosis based on established mathematical framework.
