# Supplementary material for: The Structure and Organizations of ICHD-3 Differential Diagnoses through DiffNet: A Pilot Study
Source: Diagnostics (Basel). 2022 Oct 25;12(11):2589. doi: 10.3390/diagnostics12112589 (PMC9689765; doi:10.3390/diagnostics12112589)
Supplement: Supplementary file 1 [file diagnostics-12-02589-s001.zip › diagnostics-1941791-supplementary/Table S1.pdf]

Table S1a. A list of Lower Sets

1. acute headache attributed to traumatic injury to the head
2. acute headache or facial or neck pain attributed to cervical carotid or vertebral artery dissection
3. calcitonin gene related peptide (cgrp) induced headache
4. cervicogenic headache
5. chronic cluster headache
6. chronic tension type headache
7. cluster headache
8. cold stimulus headache
9. frequent episodic tension type headache
10. headache attributed to a substance or its withdrawal
11. headache attributed to cranial and/or cervical vascular disorder
12. headache attributed to disorder of homoeostasis
13. headache attributed to epileptic seizure
14. headache attributed to increased cerebrospinal fluid (csf) pressure
15. headache attributed to infection
16. headache attributed to low cerebrospinal fluid (csf) pressure
17. headache attributed to non traumatic intracranial haemorrhage
18. headache attributed to non vascular intracranial disorder
19. headache attributed to psychiatric disorder
20. headache attributed to reversible cerebral vasoconstriction syndrome (rcvs)
21. headache attributed to spontaneous intracranial hypotension
22. headache attributed to trauma or injury to the head and/or neck
23. headache attributed to use of or exposure to a substance
24. headache or facial pain attributed to disorder of the cranium neck eyes ears nose sinuses teeth mouth or other facial or cervical structure
25. hemicrania continua
26. hemiplegic migraine
27. histamine induced headache
28. hypnic headache
29. infrequent episodic tension type headache
30. medication overuse headache (moh)
31. migraine
32. migraine with aura
33. migraine without aura
34. nitric oxide (no) donor induced headache
35. non menstrual migraine without aura
36. other headache disorders
37. other primary headache disorders
38. painful lesions of the cranial nerves and other facial pain
39. persistent headache attributed to past ischaemic stroke (cerebral infarction)
40. persistent headache attributed to past non traumatic intracranial haemorrhage
41. persistent headache attributed to traumatic injury to the head

42. primary cough headache
43. primary exercise headache
44. primary headache associated with sexual activity
45. primary stabbing headache
46. short lasting unilateral neuralgiform headache attacks
47. short lasting unilateral neuralgiform headache attacks with conjunctival injection and tearing (sunct)
48. tension type headache (alternative criteria)
49. tension type headache (tth)
50. trigeminal autonomic cephalalgias (tacs)
51. trigeminal neuralgia

Table S1b: A list of Singletons

1. acute headache attributed to moderate or severe traumatic injury to the head
2. angiography headache
3. caffeine withdrawal headache
4. cardiac cephalalgia
5. chronic migraine
6. chronic paroxysmal hemicrania
7. dialysis headache
8. diving headache
9. external compression headache
10. headache attributed to an intracranial endarterial procedure
11. headache attributed to arteriovenous malformation (avm)
12. headache attributed to cavernous angioma
13. headache attributed to cerebral autosomal dominant arteriopathy with subcortical infarcts and leukoencephalopathy (cadasil)
14. headache attributed to cerebral venous thrombosis (cvt)
15. headache attributed to chiari malformation type i (cm1)
16. headache attributed to encephalotrigeminal or leptomeningeal angiomatosis (sturge weber syndrome)
17. headache attributed to fasting
18. headache attributed to human immunodeficiency virus (hiv) infection
19. headache attributed to hypothalamic or pituitary hyper or hyposecretion
20. headache attributed to hypothyroidism
21. headache attributed to idiopathic intracranial hypertension (iih)
22. headache attributed to ingestion or inhalation of a cold stimulus
23. headache attributed to intracranial hypertension secondary to metabolic toxic or hormonal cause
24. headache attributed to long term use of non headache medication
25. headache attributed to mitochondrial encephalopathy lactic acidosis and stroke like episodes (melas)
26. headache attributed to moyamoya angiopathy (mma)
27. headache attributed to other chronic intracranial vasculopathy
28. headache attributed to transient ischaemic attack (tia)
29. migraine aura triggered seizure
30. new daily persistent headache (ndph)
31. oestrogen withdrawal headache
32. persistent headache attributed to craniotomy
33. persistent headache attributed to moderate or severe traumatic injury to the head
34. phosphodiesterase (pde) inhibitor induced headache
35. primary thunderclap headache
36. probable migraine
37. probable tension type headache
38. recurrent painful ophthalmoplegic neuropathy

39. status migrainosus
40. syndrome of transient headache and neurological deficits with cerebrospinal fluid lymphocytosis (handl)
41. triptan overuse headache
42. trochlear headache
