# Supplementary material for: The Structure and Organizations of ICHD-3 Differential Diagnoses through DiffNet: A Pilot Study
Source: Diagnostics (Basel). 2022 Oct 25;12(11):2589. doi: 10.3390/diagnostics12112589 (PMC9689765; doi:10.3390/diagnostics12112589)
Supplement: Supplementary file 1 [file diagnostics-12-02589-s001.zip › diagnostics-1941791-supplementary/Table S2.pdf]

Table S2: Lower-set Hierarchy (Table S1a, expanded)

1) acute headache attributed to traumatic injury to the head
   a) acute headache attributed to mild traumatic injury to the head
   b) acute headache attributed to whiplash
   c) delayed onset acute headache attributed to mild traumatic injury to the head
   d) delayed onset acute headache attributed to moderate or severe traumatic injury to the head

2) acute headache or facial or neck pain attributed to cervical carotid or vertebral artery dissection
   a) persistent headache or facial or neck pain attributed to past cervical carotid or vertebral artery dissection
      i) headache or facial or neck pain attributed to cervical carotid or vertebral artery dissection

3) calcitonin gene related peptide (cgrp) induced headache
   a) delayed cgrp induced headache
   b) immediate cgrp induced headache

4) cervicogenic headache
   a) headache attributed to cervical myofascial pain
   b) headache attributed to upper cervical radiculopathy

5) chronic cluster headache
   a) episodic cluster headache

6) chronic tension type headache
   a) chronic tension type headache not associated with pericranial tenderness

7) cluster headache
   a) episodic cluster headache

8) cold stimulus headache
   a) headache attributed to external application of a cold stimulus
   b) probable cold stimulus headache
      i) headache probably attributed to external application of a cold stimulus
      ii) headache probably attributed to ingestion or inhalation of a cold stimulus

9) frequent episodic tension type headache
   a) frequent episodic tension type headache not associated with pericranial tenderness

10) headache attributed to a substance or its withdrawal
   a) carbon monoxide (co) induced headache
   b) cocaine induced headache
   c) delayed alcohol induced headache
      i) alcohol induced headache
      ii) immediate alcohol induced headache
   d) delayed cgrp induced headache
   e) delayed histamine induced headache
   f) delayed no donor induced headache
   g) headache attributed to exogenous acute pressor agent
   h) headache attributed to occasional use of non headache medication
   i) headache attributed to substance withdrawal

      i) headache attributed to withdrawal from chronic use of other substance
      ii) opioid withdrawal headache
   j) headache attributed to use of or exposure to other substance
   k) immediate cgrp induced headache
   l) immediate histamine induced headache
   m) immediate no donor induced headache
   n) medication overuse headache attributed to multiple drug classes not individually overused
      i) combination analgesic overuse headache
      ii) ergotamine overuse headache
      iii) opioid overuse headache
   o) medication overuse headache attributed to other medication
   p) medication overuse headache attributed to unspecified or unverified overuse of multiple drug classes
   q) non opioid analgesic overuse headache
      i) acetylsalicylic acid overuse headache
      ii) non steroidal anti inflammatory drug (nsaid) overuse headache
      iii) other non opioid analgesic overuse headache
      iv) paracetamol (acetaminophen) overuse headache
   r) persistent headache attributed to past use of or exposure to a substance

11) headache attributed to cranial and/or cervical vascular disorder
   a) acute headache attributed to non traumatic acute subdural haemorrhage (asdh)
   b) acute headache attributed to non traumatic intracerebral haemorrhage
   c) acute headache attributed to non traumatic subarachnoid haemorrhage (sah)
   d) headache and/or migraine like aura attributed to chronic intracranial vasculopathy
      i) headache attributed to syndrome of retinal vasculopathy with cerebral leukoencephalopathy and systemic manifestations (rvclsm)
   e) headache attributed to arteritis
      i) headache attributed to giant cell arteritis (gca)
      ii) headache attributed to primary angiitis of the central nervous system (pacns)
      iii) headache attributed to secondary angiitis of the central nervous system (sacns)
   f) headache attributed to cerebral ischaemic event
      i) acute headache attributed to ischaemic stroke (cerebral infarction)
      ii) headache attributed to ischaemic stroke (cerebral infarction)
   g) headache attributed to cervical carotid or vertebral artery disorder
      i) headache attributed to carotid or vertebral angioplasty or stenting
      ii) headache or facial or neck pain attributed to cervical carotid or vertebral artery dissection
      iii) post endarterectomy headache
   h) headache attributed to cranial venous disorder
      i) headache attributed to cranial venous sinus stenting
   i) headache attributed to intracranial artery dissection
   j) headache attributed to other acute intracranial arterial disorder
      i) acute headache attributed to reversible cerebral vasoconstriction syndrome (rcvs)
         (1) acute headache probably attributed to reversible cerebral vasoconstriction syndrome (rcvs)
         (2) persistent headache attributed to past reversible cerebral vasoconstriction syndrome (rcvs)
   k) headache attributed to pituitary apoplexy

l) headache attributed to unruptured vascular malformation
   i) headache attributed to dural arteriovenous fistula (davf)
   ii) headache attributed to unruptured saccular aneurysm
m) migraine like aura attributed to cerebral amyloid angiopathy (caa)
n) persistent headache attributed to past cranial and/or cervical vascular disorder
o) persistent headache attributed to past non traumatic acute subdural haemorrhage
p) persistent headache attributed to past non traumatic intracerebral haemorrhage
q) persistent headache attributed to past non traumatic subarachnoid haemorrhage

12) headache attributed to disorder of homoeostasis
   a) head and/or neck pain attributed to orthostatic (postural) hypotension
   b) headache attributed to arterial hypertension
      i) headache attributed to autonomic dysreflexia
      ii) headache attributed to phaeochromocytoma
         (1) headache attributed to hypertensive crisis without hypertensive encephalopathy
         (2) headache attributed to hypertensive encephalopathy
      iii) headache attributed to pre eclampsia or eclampsia
   c) headache attributed to hypoxia and/or hypercapnia
      i) headache attributed to aeroplane travel
      ii) high altitude headache
      iii) sleep apnoea headache
   d) headache attributed to other disorder of homeostasis
      i) headache attributed to other metabolic or systemic disorder
      ii) headache attributed to travel in space
   e) persistent headache attributed to past disorder of homoeostasis

13) headache attributed to epileptic seizure ictal epileptic headache
   a) ictal epileptic headache
   b) post electroconvulsive therapy (ect) headache
   c) post ictal headache

14) headache attributed to increased cerebrospinal fluid (csf) pressure
   a) headache attributed to intracranial hypertension secondary to chromosomal disorder
   b) headache attributed to intracranial hypertension secondary to hydrocephalus

15) headache attributed to infection
   a) headache attributed to intracranial infection
      i) headache attributed to bacterial meningitis or meningoencephalitis
         (1) acute headache attributed to bacterial meningitis or meningoencephalitis
         (2) chronic headache attributed to bacterial meningitis or meningoencephalitis
         (3) persistent headache attributed to past bacterial meningitis or meningoencephalitis
      ii) headache attributed to intracranial fungal or other parasitic infection
         (1) acute headache attributed to intracranial fungal or other parasitic infection

        (2)   chronic headache attributed to intracranial fungal or other parasitic infection
        (3)   persistent headache attributed to past intracranial fungal or other parasitic infection
    iii) headache attributed to localized brain infection
    iv) headache attributed to viral meningitis or encephalitis
        (1)   headache attributed to viral encephalitis
        (2)   headache attributed to viral meningitis
  b) headache attributed to systemic infection
    i) headache attributed to other systemic infection
        (1)   acute headache attributed to other systemic infection
        (2)   chronic headache attributed to other systemic infection
    ii) headache attributed to systemic bacterial infection
        (1)   acute headache attributed to systemic bacterial infection
        (2)   chronic headache attributed to systemic bacterial infection
    iii) headache attributed to systemic viral infection
        (1)   acute headache attributed to systemic viral infection
        (2)   chronic headache attributed to systemic viral infection

16) headache attributed to low cerebrospinal fluid (csf) pressure
  a) post dural puncture headache

17) headache attributed to non traumatic intracranial haemorrhage
  a) persistent headache attributed to past non traumatic acute subdural haemorrhage
  b) persistent headache attributed to past non traumatic intracerebral haemorrhage
  c) persistent headache attributed to past non traumatic subarachnoid haemorrhage

18) headache attributed to non vascular intracranial disorder
  a) cerebrospinal fluid (csf) fistula headache
    i) headache attributed to intrathecal injection
  b) headache attributed to intracranial hypertension secondary to chromosomal disorder
  c) headache attributed to intracranial hypertension secondary to hydrocephalus
  d) headache attributed to intracranial neoplasia
    i) headache attributed to carcinomatous meningitis
    ii) headache attributed to colloid cyst of the third ventricle
  e) headache attributed to intracranial neoplasm
    i) headache attributed to colloid cyst of the third ventricle
  f) headache attributed to non infectious inflammatory intracranial disease
    i) headache attributed to aseptic (non infectious) meningitis
    ii) headache attributed to lymphocytic hypophysitis
    iii) headache attributed to neurosarcoidosis
    iv) headache attributed to other non infectious inflammatory intracranial disease
  g) headache attributed to other non vascular intracranial disorder
  h) ictal epileptic headache
  i) persistent headache attributed to past non vascular intracranial disorder
  j) post dural puncture headache
  k) post electroconvulsive therapy (ect) headache
  l) post ictal headache

19) headache attributed to psychiatric disorder
   a) headache attributed to depressive disorder
   b) headache attributed to generalized anxiety disorder
   c) headache attributed to panic disorder
   d) headache attributed to post traumatic stress disorder (ptsd)
   e) headache attributed to psychotic disorder
   f) headache attributed to separation anxiety disorder
   g) headache attributed to social anxiety disorder (social phobia)
   h) headache attributed to somatization disorder
   i) headache attributed to specific phobia

20) headache attributed to reversible cerebral vasoconstriction syndrome (rcvs)
   a) acute headache attributed to reversible cerebral vasoconstriction syndrome (rcvs)
   b) persistent headache attributed to past reversible cerebral vasoconstriction syndrome (rcvs)

21) headache attributed to spontaneous intracranial hypotension
   a) post dural puncture headache

22) headache attributed to trauma or injury to the head and/or neck
   a) acute headache attributed to craniotomy
   b) acute headache attributed to mild traumatic injury to the head
   c) acute headache attributed to other trauma or injury to the head and/or neck
   d) acute headache attributed to whiplash
   e) delayed onset acute headache attributed to mild traumatic injury to the head
   f) delayed onset acute headache attributed to moderate or severe traumatic injury to the head
   g) delayed onset persistent headache attributed to mild traumatic injury to the head
   h) delayed onset persistent headache attributed to moderate or severe traumatic injury to the head
   i) headache attributed to radiosurgery of the brain
   j) persistent headache attributed to mild traumatic injury to the head
   k) persistent headache attributed to other trauma or injury to the head and/or neck

23) headache attributed to use of or exposure to a substance
   a) carbon monoxide (co) induced headache
   b) cocaine induced headache
   c) delayed alcohol induced headache
      i) alcohol induced headache
      ii) immediate alcohol induced headache
   d) delayed cgrp induced headache
   e) delayed histamine induced headache
   f) delayed no donor induced headache
   g) headache attributed to exogenous acute pressor agent
   h) headache attributed to occasional use of non headache medication
   i) immediate cgrp induced headache
   j) immediate histamine induced headache
   k) immediate no donor induced headache

24) headache or facial pain attributed to disorder of the cranium neck eyes ears nose sinuses teeth mouth or other facial or cervical structure
   a) head or facial pain attributed to inflammation of the stylohyoid ligament
   b) headache attributed to disorder of cranial bone
   c) headache attributed to disorder of the ears
   d) headache attributed to disorder of the eyes
      i) headache attributed to acute angle closure glaucoma
      ii) headache attributed to ocular inflammatory disorder
      iii) headache attributed to refractive error
   e) headache attributed to disorder of the neck
      i) headache attributed to cervical myofascial pain
      ii) headache attributed to craniocervical dystonia
      iii) headache attributed to retropharyngeal tendonitis
      iv) headache attributed to upper cervical radiculopathy
   f) headache attributed to disorder of the nose or paranasal sinuses
      i) headache attributed to acute rhinosinusitis
      ii) headache attributed to chronic or recurring rhinosinusitis
      iii) headache attributed to disorder of the nasal mucosa turbinates or septum
   g) headache attributed to disorder of the teeth
   h) headache attributed to heterophoria or heterotropia
   i) headache attributed to temporomandibular disorder (tmd)
   j) headache or facial pain attributed to other disorder of cranium neck eyes ears nose sinuses teeth mouth or other facial or cervical structure

25) hemicrania continua
   a) hemicrania continua remitting subtype
   b) hemicrania continua unremitting subtype

26) hemiplegic migraine
   a) familial hemiplegic migraine (fhm)
      i) familial hemiplegic migraine other loci
      ii) familial hemiplegic migraine type 2 (fhm2)
      iii) familial hemiplegic migraine type 3 (fhm3)
   b) familial hemiplegic migraine type 1 (fhm1)
   c) sporadic hemiplegic migraine (shm)

27) histamine induced headache
   a) delayed histamine induced headache
   b) immediate histamine induced headache

28) hypnic headache
   a) probable hypnic headache

29) infrequent episodic tension type headache
   a) infrequent episodic tension type headache not associated with pericranial tenderness

30) medication overuse headache (moh)
   a) medication overuse headache attributed to multiple drug classes not individually overused
      i) combination analgesic overuse headache
      ii) ergotamine overuse headache
      iii) opioid overuse headache
   b) medication overuse headache attributed to other medication

c) medication overuse headache attributed to unspecified or unverified overuse of multiple drug classes
d) non opioid analgesic overuse headache
   i) acetylsalicylic acid overuse headache
   ii) non steroidal anti inflammatory drug (nsaid) overuse headache
   iii) other non opioid analgesic overuse headache
   iv) paracetamol (acetaminophen) overuse headache

31) migraine
   a) benign paroxysmal torticollis
   b) chronic migraine (alternative criteria)
   c) chronic migraine with continuous pain
   d) chronic migraine with pain free periods
   e) complications of migraine
   f) episodic syndromes that may be associated with migraine
      i) abdominal migraine
      ii) alternating hemiplegia of childhood
      iii) benign paroxysmal vertigo
      iv) infantile colic
      v) recurrent gastrointestinal disturbance
         (1) cyclical vomiting syndrome
   g) familial hemiplegic migraine (fhm)
      i) familial hemiplegic migraine other loci
      ii) familial hemiplegic migraine type 2 (fhm2)
      iii) familial hemiplegic migraine type 3 (fhm3)
   h) menstrually related migraine without aura
   i) migraine aura status
   j) migraine with brainstem aura
   k) migraine with typical aura
   l) migrainous infarction
   m) non menstrual migraine with aura
      i) menstrually related migraine with aura
      ii) pure menstrual migraine with aura
   n) persistent aura without infarction
   o) probable migraine with aura
   p) probable migraine without aura
   q) pure menstrual migraine without aura
   r) retinal migraine
   s) typical aura with headache
   t) typical aura without headache
   u) vestibular migraine
   v) visual snow

32) migraine with aura
   a) familial hemiplegic migraine (fhm)
         (1) familial hemiplegic migraine other loci
         (2) familial hemiplegic migraine type 2 (fhm2)
         (3) familial hemiplegic migraine type 3 (fhm3)
   b) infantile colic
   c) migraine with brainstem aura
   d) migraine with typical aura
   e) non menstrual migraine with aura
      i) menstrually related migraine with aura
      ii) pure menstrual migraine with aura
   f) probable migraine with aura

g) retinal migraine
h) typical aura with headache
i) typical aura without headache

33) migraine without aura
   a) infantile colic
   b) menstrually related migraine without aura
   c) non menstrual migraine with aura
      i) menstrually related migraine with aura
      ii) pure menstrual migraine with aura
   d) pure menstrual migraine without aura

34) nitric oxide (no) donor induced
   a) headache headache attributed to use of or exposure to other substance
   b) immediate no donor induced headache

35) non menstrual migraine without aura
   a) menstrually related migraine without aura
   b) pure menstrual migraine without aura

36) other headache disorders
   a) headache not elsewhere classified
   b) headache unspecified

37) other primary headache disorders
   a) epicrania fugax
   b) external pressure headache
      i) external traction headache
      ii) probable external pressure headache
         (1) probable external compression headache
         (2) probable external traction headache
   c) headache attributed to external application of a cold stimulus
   d) nummular headache
   e) probable cold stimulus headache
      i) headache probably attributed to external application of a cold stimulus
      ii) headache probably attributed to ingestion or inhalation of a cold stimulus
   f) probable hypnic headache
   g) probable new daily persistent headache
   h) probable nummular headache
   i) probable primary cough headache
   j) probable primary exercise headache
   k) probable primary headache associated with sexual activity
   l) probable primary stabbing headache

38) painful lesions of the cranial nerves and other facial pain
   a) glossopharyngeal neuralgia
      i) classical glossopharyngeal neuralgia
      ii) idiopathic glossopharyngeal neuralgia
      iii) secondary glossopharyngeal neuralgia
   b) headache attributed to ischaemic ocular motor nerve palsy
   c) neck tongue syndrome
   d) occipital neuralgia
   e) pain attributed to a lesion or disease of nervus intermedius
      i) nervus intermedius neuralgia

        (1) classical nervus intermedius neuralgia
        (2) idiopathic nervus intermedius neuralgia
    ii) painful nervus intermedius neuropathy
        (1) idiopathic painful nervus intermedius neuropathy
        (2) painful nervus intermedius neuropathy attributed to other disorder
        (3) post herpetic neuralgia of nervus intermedius
    iii) painful nervus intermedius neuropathy attributed to herpes zoster
        (1) post herpetic neuralgia of nervus intermedius
    iv) secondary nervus intermedius neuralgia
        (1) idiopathic nervus intermedius neuralgia
  f) pain attributed to a lesion or disease of the glossopharyngeal nerve
    i) classical glossopharyngeal neuralgia
    ii) idiopathic glossopharyngeal neuralgia
    iii) painful glossopharyngeal neuropathy
        (1) idiopathic painful glossopharyngeal neuropathy
        (2) painful glossopharyngeal neuropathy attributed to a known cause
    iv) secondary glossopharyngeal neuralgia
  g) pain attributed to a lesion or disease of the trigeminal nerve
    i) burning mouth syndrome (bms)
    ii) central neuropathic pain
    iii) central neuropathic pain attributed to multiple sclerosis (ms)
    iv) classical trigeminal neuralgia
        (1) classical trigeminal neuralgia purely paroxysmal
        (2) classical trigeminal neuralgia with concomitant continuous pain
    v) idiopathic trigeminal neuralgia
        (1) idiopathic trigeminal neuralgia with concomitant continuous pain
    vi) painful post traumatic trigeminal
        (1) neuropathy persistent idiopathic facial pain (pifp)
    vii) painful trigeminal neuropathy
        (1) idiopathic painful trigeminal neuropathy
        (2) painful trigeminal neuropathy attributed to herpes zoster
        (3) trigeminal post herpetic neuralgia
    viii) painful trigeminal neuropathy attributed to other disorder
    ix) secondary trigeminal neuralgia
        (1) trigeminal neuralgia attributed to other cause
        (2) trigeminal neuralgia attributed to space occupying lesion
    x) trigeminal neuralgia attributed to multiple sclerosis
  h) painful optic neuritis
  i) paratrigeminal oculosympathetic (raeder s) syndrome
  j) tolosa hunt syndrome

39) persistent headache attributed to past ischaemic stroke (cerebral infarction)
  a) acute headache attributed to ischaemic stroke (cerebral infarction)
  b) headache attributed to ischaemic stroke (cerebral infarction)

40) persistent headache attributed to past non traumatic intracranial haemorrhage
  a) persistent headache attributed to past non traumatic acute subdural haemorrhage

- b) persistent headache attributed to past non traumatic intracerebral haemorrhage
- c) persistent headache attributed to past non traumatic subarachnoid haemorrhage

41) persistent headache attributed to traumatic injury to the head
- a) delayed onset persistent headache attributed to mild traumatic injury to the head
- b) delayed onset persistent headache attributed to moderate or severe traumatic injury to the head
- c) persistent headache attributed to mild traumatic injury to the head
- d) persistent headache attributed to whiplash

42) primary cough headache
- a) probable primary cough headache

43) primary exercise headache
- a) probable primary exercise headache

44) primary headache associated with sexual activity
- a) probable primary headache associated with sexual activity

45) primary stabbing headache
- a) probable primary stabbing headache

46) short lasting unilateral neuralgiform headache attacks
- a) chronic sunct
- b) episodic sunct
- c) short lasting unilateral neuralgiform headache attacks with cranial autonomic symptoms (suna)
  - i) chronic suna
  - ii) episodic suna

47) short lasting unilateral neuralgiform headache attacks with conjunctival injection and tearing (sunct)
- a) episodic sunct

48) tension type headache (alternative criteria)
- a) chronic tension type headache (alternative criteria)
- b) frequent episodic tension type headache (alternative criteria)
- c) infrequent episodic tension type headache (alternative criteria)

49) tension type headache (tth)
- a) chronic tension type headache associated with pericranial tenderness
- b) chronic tension type headache not associated with pericranial tenderness
- c) frequent episodic tension type headache associated with pericranial tenderness
- d) frequent episodic tension type headache not associated with pericranial tenderness
- e) infrequent episodic tension type headache associated with pericranial tenderness
- f) infrequent episodic tension type headache not associated with pericranial tenderness
- g) probable chronic tension type headache
- h) probable frequent episodic tension type headache

    i) probable infrequent episodic tension type headache

50) trigeminal autonomic cephalalgias (tacs)
    a) chronic sunct
    b) cluster headache (alternative criteria)
    c) episodic cluster headache
    d) episodic sunct
    e) hemicrania continua (alternative criteria)
    f) hemicrania continua remitting subtype
    g) hemicrania continua unremitting subtype
    h) paroxysmal hemicrania
    i) paroxysmal hemicrania (alternative criteria)
    j) probable trigeminal autonomic cephalalgia
        i) probable cluster headache
        ii) probable hemicrania continua
        iii) probable paroxysmal hemicrania
        iv) probable short lasting unilateral neuralgiform headache attacks
    k) short lasting unilateral neuralgiform headache attacks (alternative criteria)
    l) short lasting unilateral neuralgiform headache attacks with cranial autonomic symptoms (suna)
        i) chronic suna
        ii) episodic suna
    m) undifferentiated trigeminal autonomic cephalalgia

51) trigeminal neuralgia
    a) classical trigeminal neuralgia
        i) classical trigeminal neuralgia purely paroxysmal
        ii) classical trigeminal neuralgia with concomitant continuous pain
    b) idiopathic trigeminal neuralgia idiopathic
        i) trigeminal neuralgia with concomitant continuous pain
    c) trigeminal neuralgia attributed to other cause
    d) trigeminal neuralgia attributed to space occupying lesion
