# Supplementary material for: The Structure and Organizations of ICHD-3 Differential Diagnoses through DiffNet: A Pilot Study
Source: Diagnostics (Basel). 2022 Oct 25;12(11):2589. doi: 10.3390/diagnostics12112589 (PMC9689765; doi:10.3390/diagnostics12112589)
Supplement: Supplementary file 1 [file diagnostics-12-02589-s001.zip › diagnostics-1941791-supplementary/Table S3.pdf]

Table S3: The inversions

1. acute headache attributed to reversible cerebral vasoconstriction syndrome (rcvs),persistent headache attributed to past reversible cerebral vasoconstriction syndrome (rcvs)
2. acute headache or facial or neck pain attributed to cervical carotid or vertebral artery dissection,headache or facial or neck pain attributed to cervical carotid or vertebral artery dissection
3. acute headache or facial or neck pain attributed to cervical carotid or vertebral artery dissection,persistent headache or facial or neck pain attributed to past cervical carotid or vertebral artery dissection
4. cerebrospinal fluid (csf) fistula headache,headache attributed to intrathecal injection
5. delayed alcohol induced headache,alcohol induced headache
6. delayed alcohol induced headache,immediate alcohol induced headache
7. migraine with aura,infantile colic
8. migraine without aura,infantile colic
9. painful post traumatic trigeminal neuropathy,persistent idiopathic facial pain (pifp)
10. persistent headache attributed to past ischaemic stroke (cerebral infarction),headache attributed to ischaemic stroke (cerebral infarction)
11. persistent headache or facial or neck pain attributed to past cervical carotid or vertebral artery dissection,headache or facial or neck pain attributed to cervical carotid or vertebral artery dissection
12. secondary nervus intermedius neuralgia,idiopathic nervus intermedius neuralgia
