# Supplementary material for: The Structure and Organizations of ICHD-3 Differential Diagnoses through DiffNet: A Pilot Study
Source: Diagnostics (Basel). 2022 Oct 25;12(11):2589. doi: 10.3390/diagnostics12112589 (PMC9689765; doi:10.3390/diagnostics12112589)
Supplement: Supplementary file 1 [file diagnostics-12-02589-s001.zip › diagnostics-1941791-supplementary/Table S4.pdf]

Table S4. All possible “lower bound”

acute headache attributed to traumatic injury to the head and headache attributed to trauma or injury to the head and/or neck:

- acute headache attributed to mild traumatic injury to the head
- acute headache attributed to whiplash
- delayed onset acute headache attributed to mild traumatic injury to the head
- delayed onset acute headache attributed to moderate or severe traumatic injury to the head

calcitonin gene related peptide (cgrp) induced headache and headache attributed to a substance or its withdrawal:

- delayed cgrp induced headache
- immediate cgrp induced headache

calcitonin gene related peptide (cgrp) induced headache and headache attributed to use of or exposure to a substance:

- delayed cgrp induced headache
- immediate cgrp induced headache

cervicogenic headache and headache attributed to disorder of the neck:

- headache attributed to cervical myofascial pain
- headache attributed to upper cervical radiculopathy

chronic cluster headache and cluster headache:

- episodic cluster headache

chronic cluster headache and trigeminal autonomic cephalalgias (tacs):

- episodic cluster headache

chronic tension type headache and tension type headache (tth):

- chronic tension type headache not associated with pericranial tenderness

cluster headache and trigeminal autonomic cephalalgias (tacs):

- episodic cluster headache

cold stimulus headache and other primary headache disorders:

- headache attributed to external application of a cold stimulus
- probable cold stimulus headache

episodic syndromes that may be associated with migraine and migraine with aura:

- infantile colic

episodic syndromes that may be associated with migraine and migraine without aura:

- infantile colic

frequent episodic tension type headache and tension type headache (tth):

- frequent episodic tension type headache not associated with pericranial tenderness

glossopharyngeal neuralgia and pain attributed to a lesion or disease of the glossopharyngeal nerve:

- classical glossopharyngeal neuralgia
- idiopathic glossopharyngeal neuralgia
- secondary glossopharyngeal neuralgia

headache attributed to a substance or its withdrawal and headache attributed to use of or exposure to a substance:

- carbon monoxide (co) induced headache
- cocaine induced headache
- delayed alcohol induced headache
- delayed cgrp induced headache
- delayed histamine induced headache
- delayed no donor induced headache
- headache attributed to exogenous acute pressor agent
- headache attributed to occasional use of non headache medication
- immediate cgrp induced headache
- immediate histamine induced headache
- immediate no donor induced headache

headache attributed to a substance or its withdrawal and histamine induced headache:

- delayed histamine induced headache
- immediate histamine induced headache

headache attributed to a substance or its withdrawal and medication overuse headache (moh):

- medication overuse headache attributed to multiple drug classes not individually overused
- medication overuse headache attributed to other medication
- medication overuse headache attributed to unspecified or unverified overuse of multiple drug classes
- non opioid analgesic overuse headache

headache attributed to a substance or its withdrawal and nitric oxide (no) donor induced headache:

- headache attributed to use of or exposure to other substance
- immediate no donor induced headache

headache attributed to cerebral ischaemic event and persistent headache attributed to past ischaemic stroke (cerebral infarction):

- acute headache attributed to ischaemic stroke (cerebral infarction)
- headache attributed to ischaemic stroke (cerebral infarction)

headache attributed to cervical carotid or vertebral artery disorder and persistent headache or facial or neck pain attributed to past cervical carotid or vertebral artery dissection:

- headache or facial or neck pain attributed to cervical carotid or vertebral artery dissection

headache attributed to cranial and/or cervical vascular disorder and headache attributed to non traumatic intracranial haemorrhage:

- persistent headache attributed to past non traumatic acute subdural haemorrhage
- persistent headache attributed to past non traumatic intracerebral haemorrhage
- persistent headache attributed to past non traumatic subarachnoid haemorrhage

headache attributed to cranial and/or cervical vascular disorder and persistent headache attributed to past non traumatic intracranial haemorrhage:

- persistent headache attributed to past non traumatic acute subdural haemorrhage

- persistent headache attributed to past non traumatic intracerebral haemorrhage
- persistent headache attributed to past non traumatic subarachnoid haemorrhage

headache attributed to epileptic seizure and headache attributed to non vascular intracranial disorder:

- ictal epileptic headache
- post electroconvulsive therapy (ect) headache
- post ictal headache

headache attributed to increased cerebrospinal fluid (csf) pressure and headache attributed to non vascular intracranial disorder:

- headache attributed to intracranial hypertension secondary to chromosomal disorder
- headache attributed to intracranial hypertension secondary to hydrocephalus

headache attributed to intracranial neoplasia and headache attributed to intracranial neoplasm:

- headache attributed to colloid cyst of the third ventricle

headache attributed to low cerebrospinal fluid (csf) pressure and headache attributed to non vascular intracranial disorder:

- post dural puncture headache

headache attributed to low cerebrospinal fluid (csf) pressure and headache attributed to spontaneous intracranial hypotension:

- post dural puncture headache

headache attributed to non traumatic intracranial haemorrhage and persistent headache attributed to past non traumatic intracranial haemorrhage:

- persistent headache attributed to past non traumatic acute subdural haemorrhage
- persistent headache attributed to past non traumatic intracerebral haemorrhage
- persistent headache attributed to past non traumatic subarachnoid haemorrhage

headache attributed to non vascular intracranial disorder and headache attributed to spontaneous intracranial hypotension:

- post dural puncture headache

headache attributed to other acute intracranial arterial disorder and headache attributed to reversible cerebral vasoconstriction syndrome (rcvs):

- acute headache attributed to reversible cerebral vasoconstriction syndrome (rcvs)

headache attributed to trauma or injury to the head and/or neck and persistent headache attributed to traumatic injury to the head:

- delayed onset persistent headache attributed to mild traumatic injury to the head
- delayed onset persistent headache attributed to moderate or severe traumatic injury to the head
- persistent headache attributed to mild traumatic injury to the head

headache attributed to use of or exposure to a substance and histamine induced headache:

- delayed histamine induced headache
- immediate histamine induced headache

headache attributed to use of or exposure to a substance and nitric oxide (no) donor induced headache:

- immediate no donor induced headache

hemicrania continua and trigeminal autonomic cephalalgias (tacs):

- hemicrania continua remitting subtype
- hemicrania continua unremitting subtype

hemiplegic migraine and migraine:

- familial hemiplegic migraine (fhm)

hemiplegic migraine and migraine with aura:

- familial hemiplegic migraine (fhm)

hypnic headache and other primary headache disorders:

- probable hypnic headache

infrequent episodic tension type headache and tension type headache (tth):

- infrequent episodic tension type headache not associated with pericranial tenderness

migraine and migraine with aura:

- familial hemiplegic migraine (fhm)
- migraine with brainstem aura
- migraine with typical aura
- non menstrual migraine with aura
- probable migraine with aura
- retinal migraine
- typical aura with headache
- typical aura without headache

migraine and migraine without aura:

- menstrually related migraine without aura
- non menstrual migraine with aura
- pure menstrual migraine without aura

migraine and non menstrual migraine without aura:

- menstrually related migraine without aura
- pure menstrual migraine without aura

migraine with aura and migraine without aura:

- infantile colic
- non menstrual migraine with aura

migraine without aura and non menstrual migraine without aura:

- menstrually related migraine without aura
- pure menstrual migraine without aura

nervus intermedius neuralgia and secondary nervus intermedius neuralgia:

- idiopathic nervus intermedius neuralgia

other primary headache disorders and primary cough headache:

- probable primary cough headache

other primary headache disorders and primary exercise headache:

- probable primary exercise headache

other primary headache disorders and primary headache associated with sexual activity:

- probable primary headache associated with sexual activity

other primary headache disorders and primary stabbing headache:

- probable primary stabbing headache

pain attributed to a lesion or disease of the trigeminal nerve and trigeminal neuralgia:

- classical trigeminal neuralgia
- idiopathic trigeminal neuralgia

painful nervus intermedius neuropathy and painful nervus intermedius neuropathy attributed to herpes zoster:

- post herpetic neuralgia of nervus intermedius

secondary trigeminal neuralgia and trigeminal neuralgia:

- trigeminal neuralgia attributed to other cause
- trigeminal neuralgia attributed to space occupying lesion

short lasting unilateral neuralgiform headache attacks and short lasting unilateral neuralgiform headache attacks with conjunctival injection and tearing (sunct):

- episodic sunct

short lasting unilateral neuralgiform headache attacks and trigeminal autonomic cephalalgias (tacs):

- chronic sunct
- episodic sunct
- short lasting unilateral neuralgiform headache attacks with cranial autonomic symptoms (suna)

short lasting unilateral neuralgiform headache attacks with conjunctival injection and tearing (sunct) and trigeminal autonomic cephalalgias (tacs):

- episodic sunct
