# Supplementary figures and images for: The Structure and Organizations of ICHD-3 Differential Diagnoses through DiffNet: A Pilot Study
Source: Diagnostics (Basel). 2022 Oct 25;12(11):2589. doi: 10.3390/diagnostics12112589 (PMC9689765; doi:10.3390/diagnostics12112589)

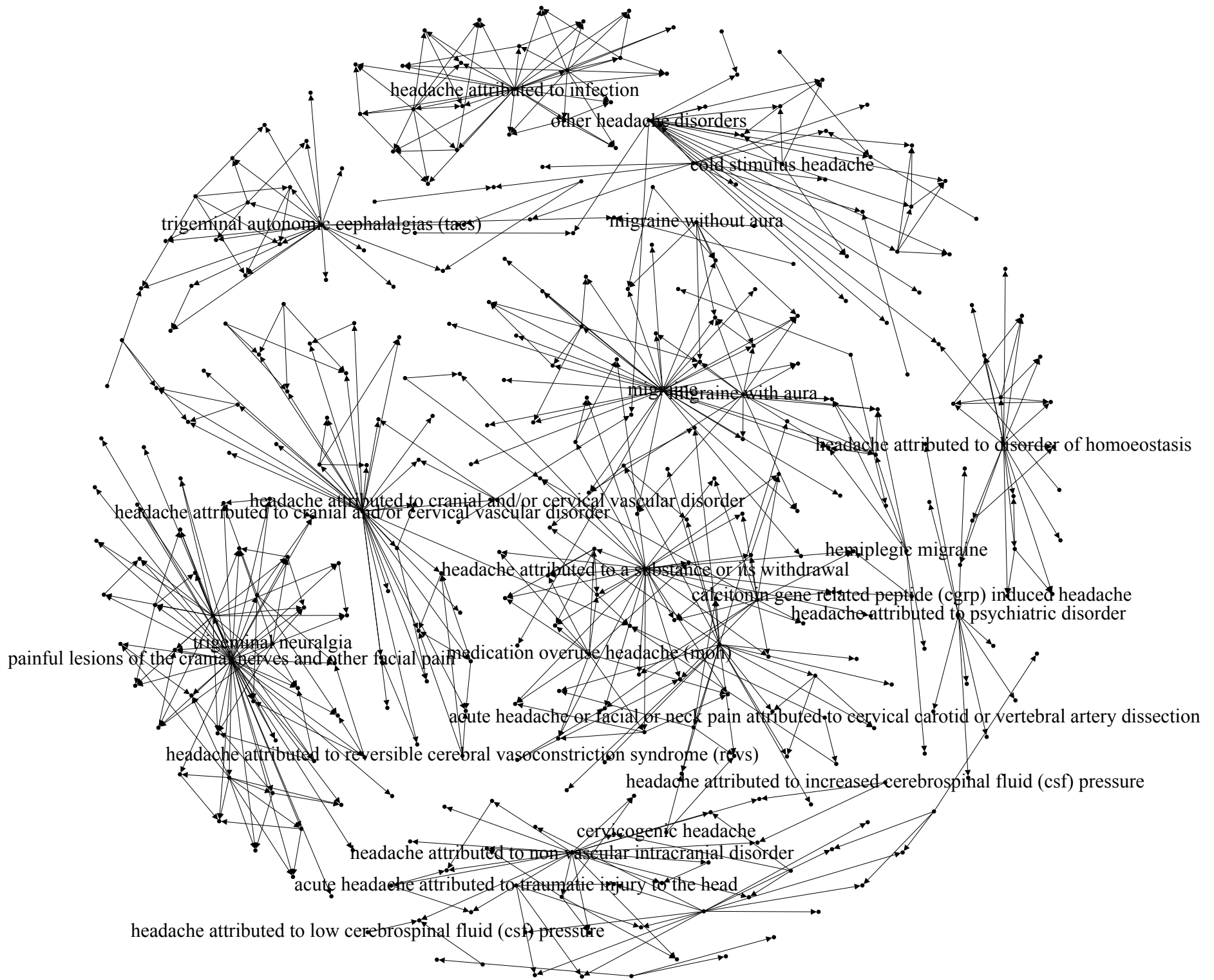

Supplement: Supplementary file 1 [file diagnostics-12-02589-s001.zip › diagnostics-1941791-supplementary/Figure S1.pdf]
